# Degradation of subchondral bone collagen in the weight-bearing area of femoral head is associated with osteoarthritis and osteonecrosis

**DOI:** 10.1186/s13018-020-02065-y

**Published:** 2020-11-11

**Authors:** Zongyi Wu, Bingzhang Wang, Jiahao Tang, Bingli Bai, Sheji Weng, Zhongjie Xie, Zijian Shen, Deyi Yan, Liang Chen, Jingdong Zhang, Lei Yang

**Affiliations:** Department of Orthopaedics Surgery, The Second Affiliated Hospital and Yuying Children’s Hospital of Wenzhou Medical University, No.109, Xueyuan West Road, Lucheng District, Wenzhou, 325000 Zhejiang, People’s Republic of China

**Keywords:** Collagen fibers, Subchondral bone, Femoral head, Osteoarthritis, Osteonecrosis

## Abstract

**Background:**

The aim of the study was to evaluate the change of subchondral bone collagen and trabecular bone in the weight-bearing area of femoral head from patients with osteoarthritis (OA) or osteonecrosis of femoral head (ONFH), and discuss the effect of collagen degradation on OA and ONFH.

**Methods:**

Femoral heads from patients with femoral neck fracture (FNF) were collected as control group. All collected samples were divided into OA group (*N* = 10), ONFH group (*N* = 10), and FNF group (*N* = 10). Differences of subchondral bone collagen were compared through scanning electron microscope (SEM) observation, immunohistochemistry staining, and Masson’s trichrome staining. Alteration of subchondral bone was displayed through hematoxylin and eosin (H&E) staining and gross morphology.

**Results:**

SEM results showed that collagen fibers in OA and ONFH group appeared to be thinner, rougher, sparser, and more wizened. Immunohistochemistry and Masson’s trichrome staining results demonstrated that the content of collagen fibers in the OA and ONFH group was obviously less than the FNF group. H&E staining results showed that trabecular bone in OA and ONFH group appeared to be thinner and ruptured. Gross morphology results showed that the degeneration and destruction of cartilage and subchondral bone in OA and ONFH group were severer than FNF group. The characteristics mentioned above in ONFH group were more apparent than OA group.

**Conclusions:**

This study revealed that degradation of collagen fibers from subchondral bone in the weight-bearing area of femoral head was associated with OA and ONFH, which may help to find new therapeutic strategies of the diseases.

## Background

Collagen fibers are the most abundant components of extracellular matrix (ECM) in connectives tissues such as bone and tendon and there is a tight equilibrium between degradation and formation of these proteins ensuring tissue health and homeostasis [1]. Abundant collagen fibrils form a three-dimensional network and provide strength as well as toughness for bone tissue, decreasing the occurrence rate of fracture and other diseases. It has been reported that trabeculae bone is full of type I collagen, and the collagen fibers are closely associated with the metabolism and biomechanics of trabeculae bone. Chipman SD et al. revealed in an animal experiment that mice with the type I collagen mutation oim present with fewer and thinner trabeculae in the femoral head compared with their wild-type counterparts [2].

Osteoarthritis (OA) is a chronic disorder, characterized by the synovial inflammation and progressive degenerative changes of cartilage [3, 4]. In healthy joints, collagen and proteoglycan are two major matrix components of articular cartilage, which function to cushion and distribute mechanical load uniformly to the whole. Articular chondrocytes maintain cartilage tissue homeostasis with a relatively low matrix turnover rate while resisting proliferation and terminal differentiation. However, at the cellular and molecular level of OA development, articular chondrocytes undergo significant alterations from their healthy homeostatic states to catabolic states. On the cellular level, OA is accompanied by proliferation of chondrocytes, hypertrophic differentiation of chondrocytes, reduction of collagen matrix deposition, remodeling and mineralization of the extracellular matrix, and apoptotic death of chondrocytes [5]. In addition, injury to the articular cartilage over time led to changes in metabolism of chondrocytes and synovial cells such that inflammatory cytokines produced impair the chondrocytes ability to recover cartilage matrix [6]. On the biochemical level, the uncontrolled production of matrix-degrading enzymes, including matrix metalloproteinases and aggrecanases, results in the destruction of cartilage matrix [7]. Moreover, the change of subchondral bone structure is also one of the characteristics of the disease [5]. Several studies have demonstrated that the changes of subchondral bone may be an important etiological element in the development of OA [8–10], and animal experiments found that morphological changes in the subchondral bone prior to articular cartilage manifestations [11–13]. Moreover, the influence of subchondral bone and articular cartilage seems to be interactive. According to the view of Mansell J P, loss of articular cartilage may alter load distribution and/or intensity of subchondral bone, which may in turn modulate certain metabolic processes as well as bone turnover [14]. Osteonecrosis of femoral head (ONFH) is a progressive disease due to decreased vascular supply to the subchondral bone of the femoral head, leading to death of osteocyte, degeneration, and necrosis of subchondral bone and eventually collapse of femoral head [15, 16]. Disruption of the mineralized collagen compromises the quality of bone tissue, leading to increased bone fragility [17, 18].Besides, articular cartilage impairment is also involved in the disease, which may be another factor that accelerate the damage of collagen fibers under articular cartilage. It can be inferred that the alteration of collagen fibers in the subchondral bone is prominent especially in the weight-bearing area of femoral head. Therefore, the impairment of subchondral bone collagen is one of the key reasons that account for the occurrence and development of OA and ONFH. Although both ONFH and OA are accompanied by subchondral bone damage, the mechanism of injury is different. In ONFH, degeneration and necrosis of subchondral bone are associated with decreased vascular supply, while in OA, subchondral bone damage may be affected by degeneration of articular cartilage and its own microfractures [19]. Consequently, there may be differences in the alteration of collagen fibers in subchondral bone tissue between ONFH and OA. A well cognition about the alteration of collagen fibers from subchondral bone in the weight-bearing area is helpful to better understand the diseases and may offer more possibility to the therapy of the diseases. Hence, the study was designed to investigate the differences of subchondral bone collagen and trabecular bone among patients with OA, ONFH, or femoral neck fracture (FNF) through scanning electron microscope (SEM) observation, immunohistochemistry staining, Masson’s trichrome staining, hematoxylin and eosin (H&E) staining, and gross morphology observation.

## Methods

### Sample collection

Femoral heads were collected from patients that underwent total hip arthroplasty (THA) because of OA, ONFH, or FNF during 2018 in our hospital. The age of the patients involved in the study ranged from 60 to 70 years old. All the samples were collected from patients diagnosed only one of the diseases mentioned above. For patients involved in the study, the bone mineral density (BMD) (T-score) of contralateral hip was between − 2.5 and − 1, which means all the participants were osteopenic. Patients diagnosed with osteoporosis were excluded. Femoral heads with previous surgeries, other diseases, or from patients with pathological fracture were also excluded from the study. After screening, a total of thirty samples were involved in the study and were marked as OA group (*N* = 10), ONFH group (*N* = 10), and FNF group (*N* = 10). The work has been carried out in accordance with the Declaration of Helsinki (1964) and was approved by the Ethics Committee of the university. (Ethical protocol number: 2017 Clinical Research Ethics Approval No.18). Written informed consents were obtained from all participants.

### Sample preparation

**Fig. 1:**
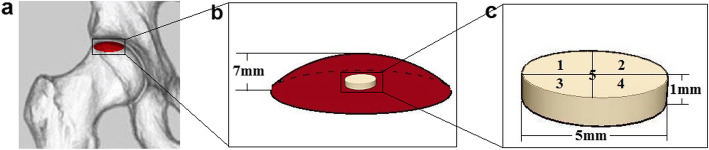
Diagrams of sample preparing procedures. **a** Area marked in red indicated weight-bearing area of femoral head. **b** Weight-bearing area measured 7 mm below the articular surface. **c** Subchondral bone obtained for testing and five SEM observation points

Samples were sent to laboratory right away after surgery and washed by deionized water to clean the blood, synovial fluid, and other tissues. Then, the samples were stored in a deep cryogenic freezer at − 80 °C. In order to obtain the subchondral bone for SEM and histology study, weight-bearing area measured 7 mm below the articular surface was sliced by band saw. Then, subchondral bone with the cartilage was obtained using a biological punch with a diameter of 5 mm. Subchondral bone with a diameter of 5 mm and a thickness of 1 mm was obtain subsequently (Fig. 1). The position of weight-bearing area and process of sample preparing was conducted based on previous studies [14, 20, 21]. Every femoral head sampled one specimen located in the center of the weight-bearing area. After that, the samples were fixed in 4% paraformaldehyde for 48 h to ensure complete tissue fixation and then decalcified in 10% ethylenediaminetetraacetic acid (EDTA); the EDTA solution was changed twice a week for 4 weeks.

### SEM observation

Before SEM observation, frozen section was performed to cut the surface produced by band saw in order to obtain an observation surface that was less of mechanical damage. After washed by phosphate-buffered saline (PBS), the samples were dehydrated in a series of graded concentration of ethanol from 70 to 100% and soaked in tert-butyl alcohol. Lyophilization was performed with a vacuum freeze drier (Christ Delta 2-24 LSC, Osterode, Germany) subsequently. Then the samples were Au sputter-coated (Eiko VX-10, Tokyo, Japan) and examined under SEM (S-3000N, HITACHI, Japan). In order to obtain more data and decrease error, five observation points were set (Fig. 1c). The diameter of collagen fibers and gap between collagen fibers were measured by ImageJ (version 1.8.0). Then, the mean values were calculated and compared in histograms.

### Immunohistochemistry staining and Masson’s trichrome staining

In order to compare the differences of collagen fibers among different groups, samples which had been decalcified in EDTA were dehydrated through a graded alcohol series and embedded in paraffin. The orientation and alignment of samples were carefully considered during paraffin embedding. Tissue sections in 6 μm thickness were mounted on glass slides and subjected to immunohistochemistry staining of type I collagen and Masson’s trichrome staining. In immunohistochemical staining, the trypsin (0.25%, T1350, Solarbio, Beijing, China) was first used to repaired antigen, and then bull serum albumin (3%, G5001, Servicebio, Wuhan, China) was used to seal tissue for 30 min. The primary antibody was rabbit anti-human collagen I antibody (1:1000, G11022-1, Servicebio, Wuhan, China). Tissue sections were incubated with first antibody overnight. Then HRP goat anti-rabbit secondary antibody (1:200, G23303, Servicebio, Wuhan, China) was added and incubated for 50 min. DAB chromogen (K5007, Dako) was used to visualize antibody labelling. In Masson’s trichrome staining, the tissue sections were first soaked in hematoxylin dye solution for 10 min, and then washed with distilled water. Then ponceau magenta dye solution was used to dip the tissue for 10 min. Finally, the tissue sections were soaked in aniline blue dye solution for 2 min. The above staining solutions were all from Masson’s trichrome staining kit (G1340, Solarbio, Shanghai, China). Examination under a microscopic light by using an Olympus DP71 microscope (Olympus Co., Japan) was held subsequently. The percentage of type I collagen in immunohistochemical staining and collagen fibers in Masson’s trichrome staining were measured by ImageJ (version 1.8.0). We set up five observation points (Fig. 1c) to reduce the error, then calculated the mean values and compared them in histograms.

### H&E staining

In order to compare the differences of trabecular bone among three groups, H&E staining was performed. Briefly, after decalcified in EDTA, samples were dehydrated through a graded alcohol series and then embedded in paraffin. Serial sections with a thickness of 4 μm were cut and mounted on polylysinecoated microscope slides. H&E staining (Solarbio, Shanghai, China) was performed subsequently according to manufacturer’s protocol and finally an examination under a microscopic light by using an Olympus DP71 microscope (Olympus Co., Japan) was held. We calculated the mean values of the percentage of trabecular bone in five observation points (Fig. 1c) with ImageJ (version 1.8.0) and compared them in histograms.

### Gross morphology of subchondral bone

In order to show the macroscopical differences of subchondral bone among the groups, gross morphology of subchondral bone was displayed. Briefly, femoral head was split in half along the coronal plane. Subchondral bone in the weight-bearing area was obtained by band saw and photographs of the subchondral bone were displayed finally.

### Statistical analysis

Baseline characteristics including age, gender, BMD (T-score), surgical side, and body mass index (BMI) among three groups were compared by SPSS software (version 18.0, by one-way ANOVA or Chi-squared Test). For SEM results, data about the diameter of collagen fibers and gap between collagen fibers was measured by ImageJ (version 1.8.0) and expressed as mean ± SD. Besides, ImageJ was used for quantitative analysis of staining results. Statistical analyses were conducted using GraphPad Prism software (version 5.0, by one-way ANOVA and Dunnett’s post hoc test or Tukey’s post hoc test). Statistical significance was considered at *p* < 0.05.

## Results

### Baseline characteristics of the patients

According to the inclusion criteria and exclusion criteria mentioned above, 10 samples were collected for each group, and a total of 30 samples (30 patients) were involved in the study. Baseline characteristics of the patients were showed in Table 1. The age in FNF group ranged from 62~70 years, and the average age was 65.4 ± 2.76 years. In OA group, the age ranged from 60~70 years, and the average age was 64.6 ± 3.10 years. In ONFH group, the age ranged from 60~69 years, and the average age was 64.9 ± 2.88 years (*p* = 0.843). There were 4 male patients and 6 female patients in FNF group. In OA group, there were 7 male patients and 3 female patients. In ONFH group, there were 6 male patients and 4 female patients (*p* = 0.531). The mean BMD (T-score) of contralateral hip according to DEXA was − 1.83 ± 0.41 (ranged from − 2.3~− 1.1) in FNF group. In OA group, the mean BMD (T-score) was − 1.78 ± 0.32 (ranged from − 2.4~− 1.4). Whereas, it was − 1.82 ± 0.40 (ranged from − 2.4~− 1.2) in ONFH group (*p* = 0.957). In FNF group, the number of left femoral head was 7 and the number of right femoral head was 3. The number was equal in OA group. In ONFH group, the number of left femoral head and right femoral head was 6 and 4, respectively (*p* = 0.893). The mean BMI in FNF group was 22.40 ± 2.55 (ranged from 19.26~25.08). In OA group, the mean BMI was 23.15 ± 2.09 (ranged from 20.18~25.30). In ONFH group, the mean BMI was 22.05 ± 2.15 (ranged from 18.94~24.21) (*p* = 0.458).

**Table 1:** Comparison of baseline characteristics in three groups

	FNF group (*n* = 10)	OA group (*n* = 10)	ONFH group (*n* = 10)	*P* value
Age	62~70 (65.4 ± 2.76)	60~70 (64.6 ± 3.10)	60~69 (64.9 ±2.88)	0.843
Gender(male/female)	4/6	7/3	6/4	0.531
BMD(T-score)	− 2.3~− 1.1 (− 1.83 ± 0.41)	− 2.4~− 1.4 (− 1.78 ± 0.32)	− 2.4~− 1.2 (− 1.82 ± 0.40)	0.957
Left/Right BMI	7/3 19.26~25.08 (22.40 ± 2.55)	5/5 20.18~25.30 (23.15 ± 2.09)	6/4 18.94~24.21 (22.05 ± 2.15)	0.893 0.458

*FNF* femoral neck fracture, *OA* osteoarthritis, *ONFH* osteonecrosis of femoral head, *BMD* bone mineral density, *BMI* body mass index

*p* < 0.05

### SEM observation

**Fig. 2:**
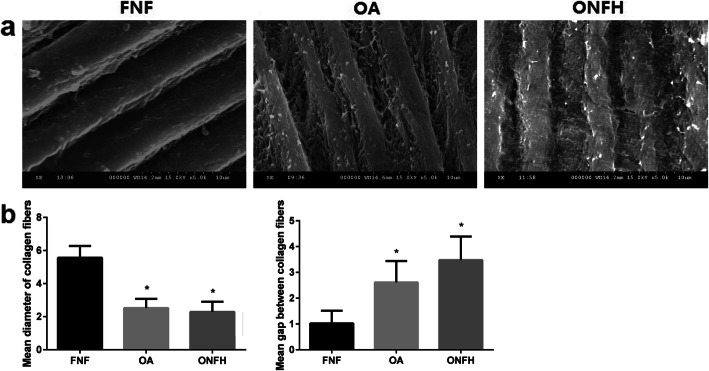
SEM results (× 5.0K) shows the differences of collagen fibers among the groups. **a** Collagen fibers in the OA and ONFH group appeared to be thinner, rougher, sparser, and more wizened compared with FNF group, and more fissures were seen on the surface of collagen fibers. The characteristics in ONFH group were more apparent than OA group. **b** Quantitative results showed the diameter of collagen fibers and gap between collagen fibers among the groups. Data were expressed as mean ± SD; *N* = 10 specimens/group. Bar = 10 μm. *p < 0.05 versus FNF group (by one-way ANOVA and Dunnett’s post hoc test)

SEM observation results clearly showed collagen fibers in the trabecular bone among the groups. Along with the orientation of trabecular bone, the arrangement of collagen fibers was parallel approximately. However, differences were observed when the observation field was further magnified. Collagen fibers in the OA and ONFH group were thinner and seemed to be more wizened, while collagen fibers in the FNF group appeared to be thicker and seemed to be plumper. The surface of collagen fibers in the OA and ONFH group was rougher compared with FNF group, and more fissures were seen on the surface of collagen fibers in the OA and ONFH group, while the surface of collagen fibers in the FNF group seemed to be smoother. The arrangement of collagen fibers in the OA and ONFH group was sparser compared with FNF group. The characteristics mentioned above in ONFH group were more apparent than OA group (Fig. 2a). Quantitative results (Fig. 2b) showed that the diameter of collagen fibers in the ONFH group was 2.3 ± 0.58 μm and it was 2.5 ± 0.51 μm in the OA group, while the diameter of collagen fibers in the FNF group was 5.6 ± 0.67 μm. The differences of OA and ONFH group were statistically significant (*p* < 0.05) compared with FNF group respectively. The gap between collagen fibers in the ONFH group was 3.5 ± 0.86 μm and it was 2.6 ± 0.78 μm in the OA group, while the gap between collagen fibers in the FNF group was 1.0 ± 0.46 μm. The differences of OA and ONFH group were statistically significant (*p* < 0.05) compared with FNF group respectively.

### Immunohistochemistry staining and Masson’s trichrome staining

**Fig. 3:**
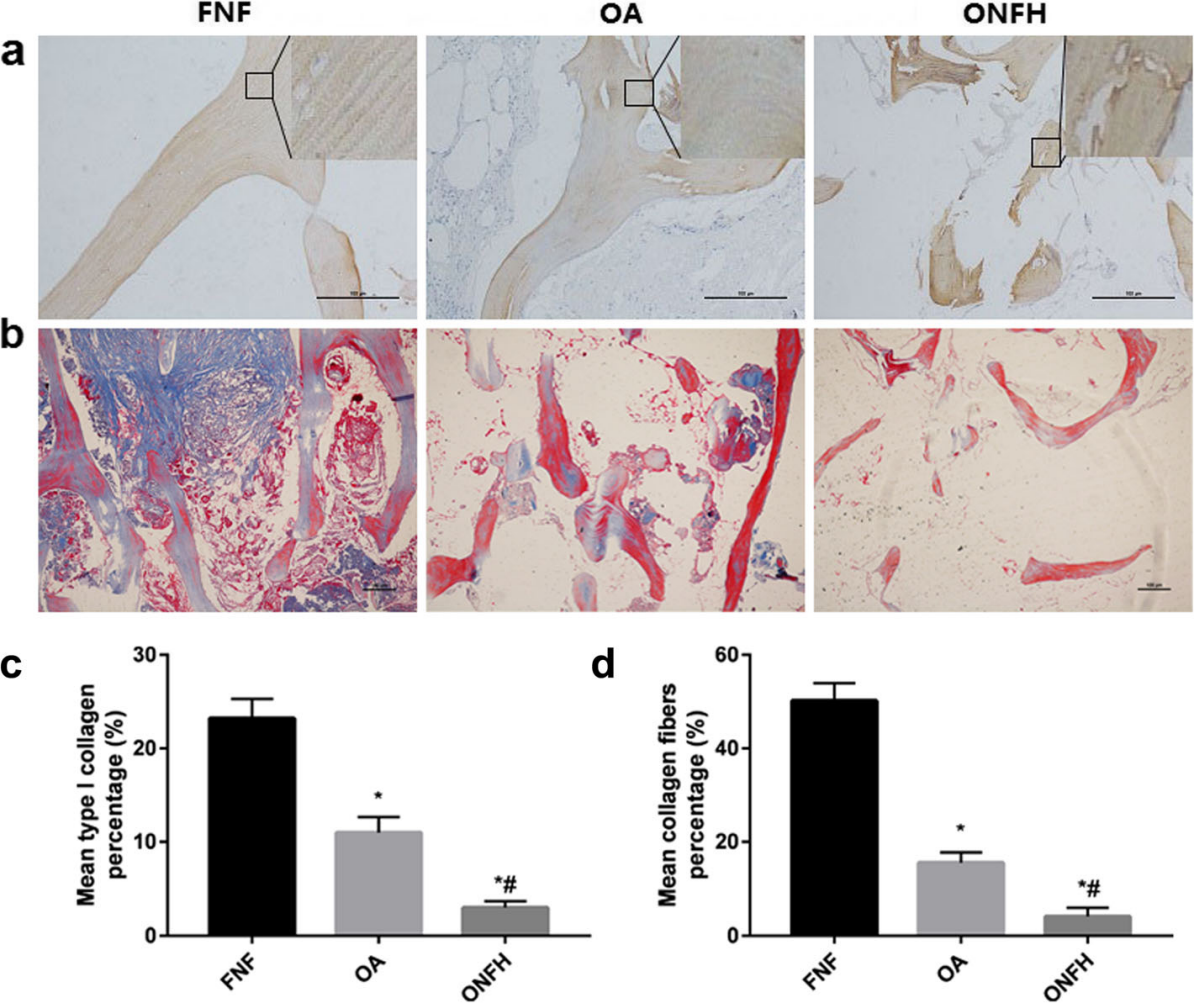
Differences of collagen staining results among the groups. **a** Immunohistochemistry staining (× 10) of type I collagen with magnifying details showed that the distribution was more uniform in the FNF group and striature result can be seen clearly. The distribution in the OA and ONFH group was less uniform. Striature result can also be seen in the OA group, but can hardly be seen in the ONFH group. Bar = 100 μm. **b** Masson’s trichrome staining (× 4) showed the blue-stained collagen fibers covered most area of the trabecular bone and striature result can be seen clearly in the FNF group. Positively stained collagen fibers in the OA group were less seen and they were sporadic in the ONFH group. Striature result can be found in the OA group, but can hardly be seen in the ONFH group. **c** Quantitative results showed the percentage of type I collagen in immunohistochemical staining among the groups. **d** Quantitative results showed the percentage of collagen fibers in Masson’s trichrome staining among the groups. Data were expressed as mean ± SD; *N* = 10 specimens/group. Bar = 100 μm. *p < 0.05 versus FNF group, #*p* < 0.05 versus OA group (by one-way ANOVA and Tukey’s post hoc test)

Immunohistochemistry staining and Masson’s trichrome staining displayed the differences of collagen fibers among the groups (Fig. 3). Immunohistochemistry staining of type I collagen with magnifying details confirmed that type I collagen distributed throughout the area of trabecular bone. The distribution in the FNF group was more uniform and striature result of type I collagen arrangement can be seen clearly. However, the distribution in the OA and ONFH group was less uniform. Striature result can also be seen in the OA group, but was not as apparent as the result in FNF group. In the ONFH group, striature result can hardly be seen and the distribution of type I collagen seemed to be most irregular among the three groups. Quantitative results (Fig. 3c) of the percentage of type I collagen in immunohistochemical staining showed that the differences of OA and ONFH group were statistically significant (*p* < 0.05) compared with FNF group respectively. The differences between OA and ONFH group were statistically significant (*p* < 0.05). Following Masson’s trichrome staining, blue-stained collagen was seen among the three groups. In the FNF group, collagen fibers covered most area of the trabecular bone and similar with immunohistochemistry staining, striature result can be seen clearly. However, positively stained collagen in the OA and ONFH group was less seen compared with FNF group. Striature staining result in the OA group still can be found but was less seen compared with FNF group. Positively stained collagen in the ONFH group appeared to be sporadic and striature result can hardly be seen. Quantitative results (Fig. 3d) showed that the percentage of collagen fibers in OA and ONFH group was less than FNF group, with statistical differences (*p* < 0.05). And there were significant differences between OA group and ONFH group (*p* < 0.05).

### H&E staining

**Fig. 4:**
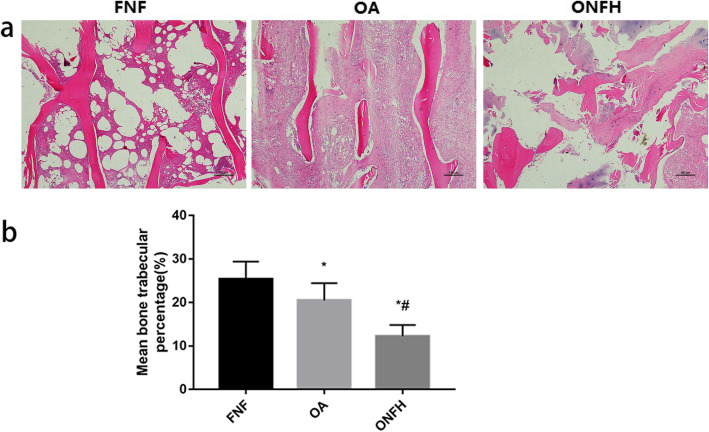
H&E staining (× 4) of trabecular bone and differences among the groups. **a** Trabecular bone in the FNF group appeared to be thicker and more integrated, the arrangement was more regular. However, trabecular bone in the OA group appeared to be slightly thinner and rupture of trabecular bone can be seen but was not quite common. Arrangement of trabecular bone in the ONFH group appeared to be disorganized and rupture was widespread. **b** Quantitative results showed the percentage of trabecular bone in H&E staining among the groups. Data were expressed as mean ± SD; *N* = 10 specimens/group. Bar = 100 μm. *p < 0.05 versus FNF group, #*p* < 0.05 versus OA group (by one-way ANOVA and Tukey’s post hoc test)

H&E staining results clearly displayed the differences of trabecular bone among the three groups (Fig. 4). It was obvious that trabecular bone in the FNF group appeared to be thicker and more integrated, and the arrangement was more regular. Trabecular bone in the OA group appeared to be slightly thinner than FNF group. Rupture of trabecular bone can be seen but was not quite common. In the ONFH group, the arrangement of trabecular bone appeared to be disorganized and rupture of trabecular bone was widespread. Quantitative results (Fig. 4b) showed that the percentage of trabecular bone in FNF group was larger than OA and ONFH group, with significant differences (*p* < 0.05). Meanwhile, the differences between OA and ONFH group were statistically significant (*p* < 0.05).

### Gross morphology of subchondral bone

**Fig. 5:**
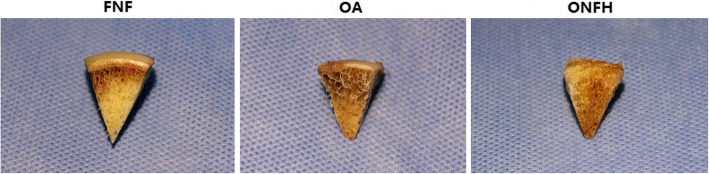
Gross morphology of subchondral bone and cartilage of the groups. Subchondral bone in the FNF group appeared to be compact and integrated. Destruction of trabecular bone was seen in the OA group, but the structure of trabecular bone arrangement was still reserved. In the ONFH group, the destruction of subchondral bone was severest, and the arrangement of trabecular bone appeared to be structureless. Cartilage in the FNF group was thicker, while it was thinner in the OA group with part of region destroyed. Cartilage in the ONFH group was thinnest and the damage was severest. Bar = 5 mm

Gross morphology of subchondral bone in the FNF group appeared to be compact and integrated. However, alteration of the structure appeared in the OA and ONFH group. Although the destruction of trabecular bone was seen in the OA group, the structure of trabecular bone arrangement was still reserved. In the ONFH group, the destruction of subchondral bone was severest, and the arrangement of trabecular bone appeared to be structureless. Besides, cartilage in the FNF group was thicker, while it was thinner in the OA group with part of region destroyed. Cartilage in the ONFH group was thinnest and the damage was severest (Fig. 5).

## Discussion

Type I collagen, as a structural protein, is the major organic component in bone matrix [22]. Collagen fibers provide a stable template for mineralization, and are important to the specific structure formation of bone. The matrices properties of collagen fibers such as tensile strength and viscoelasticity are indispensable for bone to better exert biological function. The reduction of subchondral bone collagen of femoral head may indicate an increased risk of facture as well as cartilage degeneration, leading to OA and ONFH. Besides the mechanical effect, collagen fibers were found to participate in some biochemistry and cytobiology process, influencing the metabolism of bone. Boraschi-Diaz et al. reported that osteoclast differentiation from murine bone marrow precursor is affected by type I collagen and its degradation products. Besides, the effect was found to be dose-dependent [23]. Procollagen type I N-terminal propeptide (P1NP) and serum C-terminal telopeptide of type I collagen (s-CTX) are two serum indexes indicating the condition of bone formation and bone resorption respectively. The International Osteoporosis Foundation (IOF) and the International Federation of Clinical Chemistry and Laboratory Medicine (IFCC) recommend the use of P1NP and s-CTX as reference analytes for bone turnover markers in clinical studies [24], which also reveal the relationship of collagen fibers and osteoporosis. Thus, the formation and degradation of collagen fibers may affect the occurrence and development of some diseases. Understanding the alteration of collagen fibers in specific diseases may offer new therapeutic strategies.

In this study, differences of collagen fibers from subchondral bone in the weight-bearing area of femoral head from patients with OA, ONFH, or FNF were compared. Collection of femoral head could not be finished in a short time because of the inclusion criteria and exclusion criteria. So the samples were stored in a deep cryogenic freezer at − 80 °C for batch processing. Now that all samples have been freeze processed, the influence of the process on collagen between three groups can be ignored. Similarly, in order to avoid the effect of osteoporosis, all patients involved in the study were osteopenic (− 2.5<T-score<− 1). The results of the study were displayed in the order from microcosmic to macroscopical to show the relationship of collagen fibers alteration and the diseases. SEM observation was performed primarily. Then, immunohistochemical staining and Masson’s trichrome staining were performed. Differences of collagen fibers were compared based on the experiments mentioned above. H&E staining was carried out to display the differences of trabecular bone subsequently. Finally, gross morphology of the samples was shown to display the macroscopical differences of the femoral heads. SEM results found that collagen fibers in the OA and ONFH group appeared to be thinner, rougher, sparser, and more wizened. The change in ONFH group was more apparent than OA group, which correspond to the fact that the damage of subchondral bone was usually severer in ONFH patients than OA patients. The degradation of collagen fibers in the weight-bearing area decreased the mechanical strength of subchondral bone. Large necrosis and collapse of subchondral bone may eventually lead to secondary osteoarthritis [25, 26]. Furthermore, it has been reported that the biomarkers of the bone remodeling changed at the early preclinical stage of ONFH and correlated with the severity of the pathological process [27]. Bone remodeling is necessary to maintain bone homeostasis through resorption of damaged bone and subsequent replacement of new bone [28]. Therefore, bone remodeling is regarded as one of the approaches to repair the damaged subchondral bone in patients with ONFH. The degradation products of collagen fibers were found to inhibit the differentiation of osteoclast [23], which participates in the bone resorption, and therefore, lead to the damage of bone remodeling and progress of ONFH. The results in OA group of our study were coincident with previous report that the metabolism of collagen fibers is increased within osteoarthritic femoral heads, especially within the subchondral zone. Besides, Blair-Levy J M et al. reported that altered architecture of the underlying subchondral bone may lead to progressive destruction of articular cartilage and defect of type I collagen would lead to rapidly progressive osteoarthritis [29]. The degradation of collagen fibers in OA patients was also verified in biochemical method that matrix metalloproteinase-2(MMP-2) was found increased in the subchondral bone of osteoarthritic hips. MMP-2 has been described as a marker of collagen degradation and remodeling in several connective tissues [30–32]. Moreover, considerable evidence indicates the presence of channels and fissures between cartilage and bone, providing a route for biologic signals between these compartments [33–35]. Therefore, subchondral bone and cartilage should be considered as an interdependent functional unit. Increased metabolism of collagen fibers leads to an imbalance between collagen and noncollagen protein synthesis, and then an increase of bone volume without a concomitant increase of bone mineralization, which result in a decrease of bone quality.

Now that the degradation of collagen fibers is closely related to the happen and progress of OA and ONFH. Studies focus on the prevention of collagen degradation and facilitation of collagen regeneration may be of great significance. Icaritin was found to inhibit collagen degradation-related factors and facilitate collagen accumulation [36]. And fibroblast growth factor 18 (FGF18) was found to increase collagen deposition [37]. Genes such as mothers against decapentaplegic homolog (Smad) family (Smad1-4), bone morphogenetic proteins and their receptors (Bmp2, Bmp3, Bmpr1α, and Bmpr1β), and matrix metalloproteinases (MMP2, -9, and-10) are associated with collagen biology, transcriptional control, and bone mineral metabolism, which may be the therapeutic target of collagen degradation [38]. Stem cell transplantation is another meaningful technique that is expected to serve as a new method in the treatment of ONFH [39]. Whether the technique is also valuable in the treatment of collagen degradation and the possible mechanism needs further in-depth study.

## Conclusion

Collagen fibers provide a stable template for mineralization and mechanical support of subchondral bone and take part in some biochemistry and cytobiology process, influencing the metabolism of bone. The Degradation of collagen fibers from subchondral bone in the weight-bearing area of femoral head leads to the destruction of trabecular bone, even the collapse of subchondral bone, which was associated with the occurrence and progress of OA and ONFH. Studies focus on the prevention of collagen degradation and facilitation of collagen regeneration may promote the prophylaxis and treatment of OA and ONFH.

## Data Availability

The data and materials supporting the conclusions of this article are included within the article.

## Abbreviations

OA: Osteoarthritis
ONFH: Osteonecrosis of femoral head
FNF: Femoral neck fracture
SEM: Scanning electron microscope
H&E: Hematoxylin and eosin
ECM: Extracellular matrix
THA: Total hip arthroplasty
BMD: Bone mineral density
EDTA: Ethylenediaminetetraacetic acid
PBS: Phosphate-buffered saline
BMI: Body mass index
P1NP: Procollagen type I N-terminal propeptide
s-CTX: Serum C-terminal telopeptide of type I collagen
IOF: International Osteoporosis Foundation
IFCC: International Federation of Clinical Chemistry and Laboratory Medicine
MMP-2: Matrix metalloproteinase-2
FGF18: Fibroblast growth factor 18
